# Reversible magnetism switching of iron oxide nanoparticle dispersions by controlled agglomeration†

**DOI:** 10.1039/d1na00159k

**Published:** 2021-03-30

**Authors:** Stephan Müssig, Björn Kuttich, Florian Fidler, Daniel Haddad, Susanne Wintzheimer, Tobias Kraus, Karl Mandel

**Affiliations:** Department of Chemistry and Pharmacy, Friedrich-Alexander University Erlangen-Nürnberg (FAU) Egerlandstraße 1 91058 Erlangen Germany karl.mandel@fau.de; INM – Leibniz-Institute for New Materials Campus D2 2 66123 Saarbrücken Germany tobias.kraus@leibniz-inm.de; Magnetic Resonance and X-ray Imaging Department, Development Center X-ray Technology, Fraunhofer Institute for Integrated Circuits IIS Am Hubland D-97074 Würzburg Germany; Colloid and Interface Chemistry, Saarland University 66123 Saarbrücken Germany; Fraunhofer-Institute for Silicate Research ISC Neunerplatz 2 97082 Würzburg Germany

## Abstract

The controlled agglomeration of superparamagnetic iron oxide nanoparticles (SPIONs) was used to rapidly switch their magnetic properties. Small-angle X-ray scattering (SAXS) and dynamic light scattering showed that tailored iron oxide nanoparticles with phase-changing organic ligand shells agglomerate at temperatures between 5 °C and 20 °C. We observed the concurrent change in magnetic properties using magnetic particle spectroscopy (MPS) with a temporal resolution on the order of seconds and found reversible switching of magnetic properties of SPIONs by changing their agglomeration state. The non-linear correlation between magnetization amplitude from MPS and agglomeration degree from SAXS data indicated that the agglomerates' size distribution affected magnetic properties.

## Abstract 2

Defined agglomeration of iron oxide nanoparticle dispersions was induced by temperature. The changes were characterized by magnetic particle spectroscopy *in situ* and revealed that the agglomerates' size distribution affected magnetic properties.

## Graphical abstract

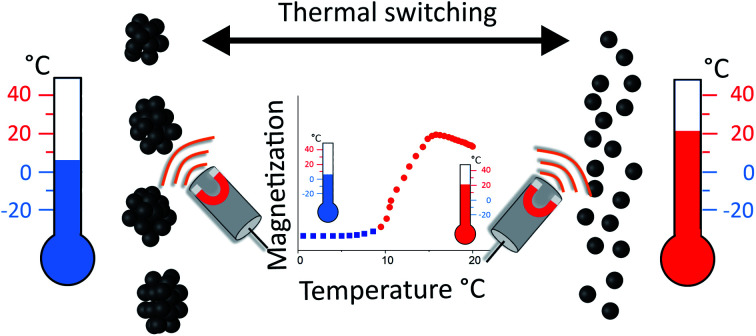

## Introduction

The agglomeration of noble metal and semiconductor nanoparticles dispersed in non-polar solvents has recently been studied in detail.^1–5^ Such non-polar particles are not dominated by electrostatic interactions. Their colloidal state depends on van der Waals attraction, steric repulsion and entropic effects of the solvent/ligand interaction that have compatible magnitudes and which standard DLVO theory does not consider. Progress in the simulation of non-polar nanoparticles beyond DLVO modelling and the preparation of highly defined non-polar particles has made it possible to curtail dispersions such that agglomeration can be induced *via* changes in temperature, particle concentration or solvent composition. The onset of agglomeration can now be adjusted by tuning particle size, ligand properties (length, coverage, branching) and the solvent.^2,3,5^ Certain types of agglomeration, for example the temperature-dependent agglomeration of alkylthiol-coated gold nanoparticles, have been shown to be fully reversible.^2,3^

Magnetic, non-polar nanoparticles add directed forces to the interactions named above. Despite of interesting fundamental and applied implications, the reversible agglomeration of non-polar magnetic nanoparticles is an unexplored research field. Recent work indicated that magnetic particle spectroscopy (MPS) can sensitively detect changes induced by agglomeration of individual magnetic nanoparticles.^6–8^ Due to the method's fast measurement speed and easily configurable measurement setup, it is potentially promising to *in situ* gain a better understanding of dynamically agglomerating magnetic particles in dispersion.^9^ With respect to future applications, being able to precisely tailor agglomeration and thus magnetic properties of nanoparticles could be utilized to improve the efficiency of hyperthermia.^10–12^ This work does not provide a final solution for medical and other applications that require aqueous particle dispersions. The aim here is to demonstrate a principle that can be transferred to aqueous systems.

The influence of temperature on the magnetization of nanoparticles has been utilized to measure the sample temperature (thermometry) by MPS.^13–15^ To acquire 2D images by nanoparticle temperature imaging or magnetic particle imaging, thermometry was successfully combined with a spatially scanning setup.^16–18^ Different magnetic properties at different temperatures yielded a high contrast, indicating the need to design nanoparticles that change their magnetization even more rapidly and strongly near room temperature.^19,20^

In this work, we studied the change of magnetic properties in superparamagnetic iron oxide nanoparticles (SPIONs) upon thermally induced agglomeration. To this end, iron oxide cores were coated with stearic acid shells in a ligand exchange step. This shell is expected to undergo temperature-dependent phase transitions which are likely to affect colloidal stability.^21^ Small-angle X-ray scattering and dynamic light scattering were then used in order to observe the temperature-dependent agglomeration and its reversibility. Our results indicate that agglomeration is reversible and switchable. We analyzed the effect of agglomeration on magnetic properties of the dispersion by magnetic particle spectroscopy (MPS). Samples with temperature-sensitive shells and reference samples that remained dispersed were characterized by *in situ* MPS during temperature variation between 25 °C and −10 °C. Thermally induced agglomeration of the SPIONs clearly altered their magnetic properties. Finally, magnetic properties were correlated with the agglomeration fraction determined by SAXS, which revealed a non-linear relationship that was affected by the structure of the agglomerates.

## Experimental

### Synthesis and stabilization of iron oxide nanoparticles with different ligand shells

Superparamagnetic iron oxide nanoparticles (SPIONs) were obtained in a continuous precipitation process by dissolving FeCl_3_·6H_2_O (10.80 g, 40 mmol, Sigma Aldrich, >99%) and FeCl_2_·4H_2_O (3.98 g, 20 mmol, Sigma Aldrich, >97%) in deionized water (125 mL) at room temperature. The liquid was mixed with 5 wt% aqueous ammonia solution NH_3_ (125 mL) by pumping the two fluids with a peristaltic pump (Ismatec MCP, flow rate: 1000 mL min^−1^) through a static mixer (plastic spiral bell mixer 7700924, Nordson Deutschland GmbH). The black precipitate was magnetically separated after 1 min and washed three times with deionized water. Half of the batch was redispersed in deionized water (125 mL) and oleic acid (3.2 g, ∼11 mmol, Sigma Aldrich) was added dropwise over 5 min while rigorously stirring the liquid for 1 h. The precipitate was magnetically separated and washed with ethanol three times. After the fourth decantation, the precipitate was redispersed in toluene to obtain a stable ferrofluid.

Ligand exchange was then performed on the oleic acid stabilized particles. In a 50 mL glass vial, 15 mL of nanoparticles dispersed in toluene were heated until the solvent evaporated completely. 25 mL of dibenzyl ether (Sigma Aldrich) was added. The solution was heated to 250 °C until redispersion, then 1 g stearic acid was added. Afterwards the heating was turned off and the reaction mixture was left on the heating plate to cool down. The reaction mixture was precipitated by adding ethanol and magnetic separation. The suspension was centrifuged for 5 min at 4000 rpm. After disposing of the supernatant, the SPIONs were redispersed in the desired solvent with a concentration of 5 wt% (SPIONs/total mass).

To show complete reversibility of agglomeration and de-agglomeration, smaller, less polydisperse SPIONs were synthesized as described in previous work.^22^ After heating 0.705 g iron(iii)acetylacetonate Fe(acac) in 20 mL oleylamine to 250 °C over 20 min, the SPIONs were decanted and redispersed in toluene. Ligand exchange to behenic acid was performed subsequently in analogy to the protocol described above.

### Transmission electron microscopy

Transmission electron microscopy (TEM) images were taken on a JEOL JEM-2011 instrument with an acceleration voltage of 200 kV. The TEM samples were prepared by drying a droplet of 2.5 μL of the particle suspension on a carbon coated copper grid.

### Raman spectroscopy

Raman spectra of the SPIONs were recorded with an inVia Raman microscope by Renishaw. Dry samples were measured after complete evaporation of the solvent at an excitation wavelength of 633 nm.

### Thermogravimetric analysis

The density of ligands on the nanoparticle surface was determined by thermogravimetric analysis (TGA). A nanoparticle dispersion was successively filled into a TGA crucible and the solvent was fully evaporated by heating the crucible to 80 °C on a hot plate. The solid sample was then heated to 1000 °C under argon atmosphere by Netzsch STA 449 F3 TGA at a heating rate of 5 K min^−1^. At the maximum temperature the furnace gas was switched to synthetic air for complete oxidation of all organic parts. Sample weight was monitored during the complete process.

### Small-angle X-ray scattering

Small-angle X-ray scattering was performed on a Xeuss 2.0 laboratory beamline by Xenocs, Grenoble, France. The instrument was equipped with a Genix3D X-ray source working on the copper Kα-line with a wavelength of *λ* = 1.54 Å, focused by a multilayer mirror and two times collimated. Scattered intensity was detected by a Pilatus3R 1M detector from Dectris, Baden, Switzerland. The sample to detector distance of 2.5 m was calibrated by using diffraction peaks from silver behenate. The accessible range of the modulus of the scattering vector (
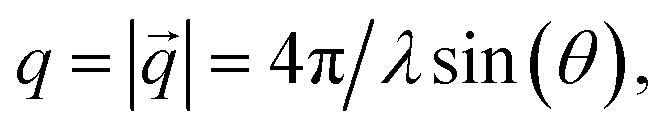
 with 2*θ* being the scattering angle), was 0.005 Å^−1^ ≤ *q* ≤ 0.24 Å^−1^. Since all samples scattered isotropically, 2D detector images were radially averaged into 1D scattering curves. Samples were filled into borosilicate glass capillaries with a diameter of 1.5 mm and sealed by epoxy glue. For temperature scans samples were cooled from 40 °C to −20 °C in steps of 2 K. Samples were equilibrated for five minutes at each step and the scattered intensity was recorded for five minutes subsequently. Heating was performed analogously after reaching −20 °C until the sample had reached 40 °C again.

The behenic acid sample was measured from 70° to −20 °C and back in steps of 10 K with 30 min equilibration time.

### Dynamic light scattering

Dynamic light scattering was performed with a Malvern Instruments (Worcestershire, UK) Zetasizer Nano ZS ZEN 3600 in a quartz cuvette at different temperatures. The dispersions were appropriately diluted and cooled to the respective temperature over 5 min. Data analysis was performed on the volume weighted hydrodynamic diameter distribution.

### Magnetic particle spectroscopy

Magnetic particle spectroscopy (MPS) measurements were performed with a self-built spectrometer based on the electronics of a commercial magnetic particle spectrometer (MPS-Unit, Pure Devices GmbH, Rimpar, Germany) and a probehead designed similar to [Patent WO2014068303]. Excitation field strength was 17 mT at a working frequency of 20 kHz. Before each measurement, calibration without probe was done, consisting of zero adjustment of the residual signal at the excitation frequency and recording of an empty spectrum that was subtracted automatically from the consecutive measured spectra. 500 μL dispersion with 5 wt% SPIONs was used in a tube with 10 mm diameter. The sample was dispersed by ultrasound with a Bandelin sonorex super RK31 for 5 min at room temperature. Cooled air was generated by pumping air through a liquid nitrogen cooled volume in a styrofoam box. The sample was cooled by this air flow with cooling rates in the order of 2–5 K min^−1^ while measuring. Reheating the sample occurred in ambient air at room temperature at <5 K min^−1^. Such small heating rates assured thermodynamic equilibrium. Measurements were performed every second during cooling and subsequent heating. One plotted data point represents 10 averaged measurements. The corresponding temperature of each data point was averaged from start and end-temperature of all measurements. The analyzed harmonics (3rd in the manuscript, higher harmonics in ESI†) were exported from every measurement and normalized with respect to the fundamental amplitude intensity of the first measurement performed at room temperature, as this also compensates for temperature-induced errors of the pick-up coil.

## Results and discussion

Temperature induced agglomeration of non-polar metal nanoparticles can be either core or shell dominated.^2,3^ Strong attractive interactions between the cores in core-dominated agglomeration overcome the steric repulsion of the ligand shell and drive agglomeration independent of the molecular conformation of the shell. Smaller cores or thicker ligand shells reduce the interactions of the metal cores and increase the relative importance of ligand shell interactions. Agglomeration in the shell dominated regime can be caused by an order–disorder phase transition of the ligand shell. The temperature of this phase transition depends on the molecular structure of the ligand molecules. Here, we study shell-dominated nanoparticles with long linear ligands (stearic acid) that undergo a phase transition and cause agglomeration around *T*_P_ ≈ 5 °C and nanoparticles with a kinked ligand (oleic acid) that remains disordered at low temperatures and keeps the particles stable to *T*_P_ < −20 °C.

Reference SPIONs were synthesized by attaching oleic acid to the surface of particles. Temperature-sensitive SPIONs were prepared by exchanging the oleic acid with stearic acid (see ESI Fig. S1† for transmission electron microscopy (TEM) micrographs). Raman spectroscopy (ESI Fig. S2†) showed no signs of oleic acid after ligand exchange, and the resulting SPIONs behaved as a stable ferrofluid. Thermogravimetric analyses (ESI Fig. S3†) revealed that the mass loading of oleic acid on the SPIONs before ligand exchange was similar to the mass loading of stearic acid after ligand exchange (81% compared to 85% residual mass). Thus, we believe that no double layer of ligands was created but rather a change of ligands occurred.

### Small-angle X-ray scattering and dynamic light scattering characterization

**Fig. 1:**
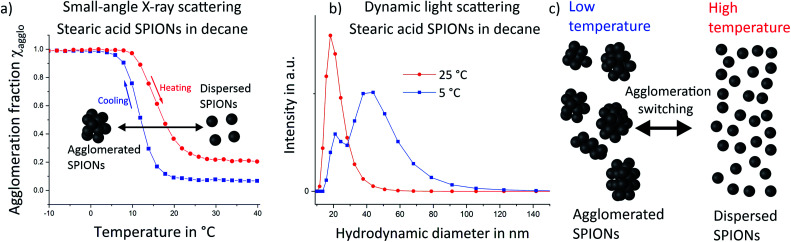
Small-angle X-ray scattering (SAXS, a) and dynamic light scattering (b) were used to quantify the temperature dependent fraction of agglomerated SPIONs in decane. Cooling to low temperatures (blue squares) led to agglomeration and high agglomeration fractions. Heating (red circles) induced de-agglomeration, with a notable hysteresis towards higher temperatures. Dynamic light scattering (the volume weighted size distribution is shown) at 5 °C (blue squares) indicated an increasing fraction of agglomerates compared to 25 °C (red circles) as schematically illustrated in (c).

Temperature dependent small-angle X-ray scattering (SAXS) was performed to follow the agglomeration process *in situ*. The scattering signal at high temperatures was dominated by the particle form factor, *i.e.* by scattering from single, well dispersed particles. A strong structure factor emerged below 20 °C (see Fig. S4 in the ESI†). Evaluation of the structure factor contribution at constant temperatures provided us with the volume fraction of particles, which are part of an agglomerate, the so-called agglomeration fraction *χ*, for each temperature (a detailed explanation is given in the ESI†). Values of the changing agglomeration fractions during a cooling and a heating run are shown in Fig. 1a. Almost all particles were well dispersed at the beginning of the cooling cycle at 40 °C (blue squares), with few agglomerates visible. During cooling, agglomeration started just below 20 °C. Most particles had agglomerated at around 5 °C (agglomeration fraction *χ* = 0.97). Heating from −20 °C (red circles) led to de-agglomeration, with a hysteretic shift of the temperature required for both the onset and complete de-agglomeration. Such shifts have been reported for noble metal and semiconductor nanoparticles before.^2,23^ Agglomeration was not fully reversed at the highest investigated temperatures of 40 °C, where approximately 20% of particles remained agglomerated; this partial irreversibility was most probably caused by larger or insufficiently functionalized nanoparticles. Complete reversibility was achieved upon using smaller and less polydisperse iron oxide nanoparticles that we prepared as described in previous work.^22^ We used temperature-dependent SAXS to follow the agglomeration of these samples and found no sign of remaining agglomerates (ESI Fig. S5†).

We confirmed the increase of agglomeration fraction upon cooling by measuring the hydrodynamic diameter distribution in the dispersion *via* dynamic light scattering (DLS) (Fig. 1b). At 25 °C (red circles), DLS indicated a narrow size distribution with a maximum around 18 nm, consistent with well-dispersed SPIONs. Note that DLS is highly sensitive to small fractions of (strongly scattering) agglomerates, which implies a very low level of agglomeration at 25 °C. Cooling the sample to 5 °C led to bimodal size distributions with one maximum at 18 nm that corresponds to individual nanoparticles. We attribute the second, broader maximum up to around 120 nm to agglomerated SPIONs. Note that quantification of agglomeration fractions from DLS data is difficult because DLS does not provide linear data on the fraction of different particle sizes. Thus, DLS measurements confirmed that agglomerates consisting of multiple SPIONs exist at low temperatures.

Both, SAXS and DLS, two complementary techniques, indicated that stearic acid-functionalized SPIONs act as a thermally switchable system in which agglomeration changes from fully dispersed at room temperature towards larger fractions of agglomerates at lower temperatures as schematically illustrated in Fig. 1c.

**Fig. 2:**
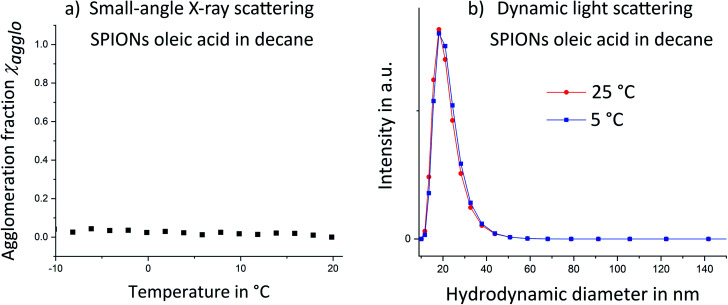
Temperature dependent small-angle X-ray scattering (SAXS) (a) and dynamic light scattering (b) showed no signs of temperature induced agglomeration for SPIONs capped with oleic acid.

As a reference system, SPIONs functionalized with oleic acid surface groups were studied. Oleic acid imparted good colloidal stability to the iron oxide cores and prevented temperature-induced agglomeration. Temperature dependent small-angle X-ray scattering (SAXS) was performed on oleic acid-covered SPIONs. No sign of agglomeration was visible down to a temperature of −20 °C (Fig. 2a; structure factors are given in Fig. S6 in the ESI†). The hydrodynamic diameter from DLS (Fig. 2b) remained almost identical when the particles were cooled from 25 °C to 5 °C. We conclude that the utilized oleic acid functionalized SPIONs stay dispersed over the analyzed temperature range.

### Magnetic characterization of non-agglomerating SPIONs

Magnetic properties of the dispersions at different stages of agglomeration were measured *via* magnetic particle spectroscopy (MPS). MPS is a recent magnetic measurement technique which detects the non-linear magnetic behavior of powder or liquid samples as explained in previous publications.^6–8,13^ Briefly, an alternating magnetic field (±17 mT) is applied and the time-dependent magnetization of the sample is detected by measuring the induced voltage in pickup coils. After fast Fourier transform (FFT), higher harmonics are obtained from the non-linear magnetization of SPIONs. Measurements require a few seconds and do not necessitate cryogenic refrigeration as in SQUID magnetometers. For this work, a setup was built that enabled *in situ* MPS during temperature variation. The measured magnetic properties were represented by plotting the relative magnetization amplitude as function of higher harmonics (Fig. S7 in ESI† shows such curves for non-agglomerating SPIONs at 25 °C and 0 °C).

**Fig. 3:**
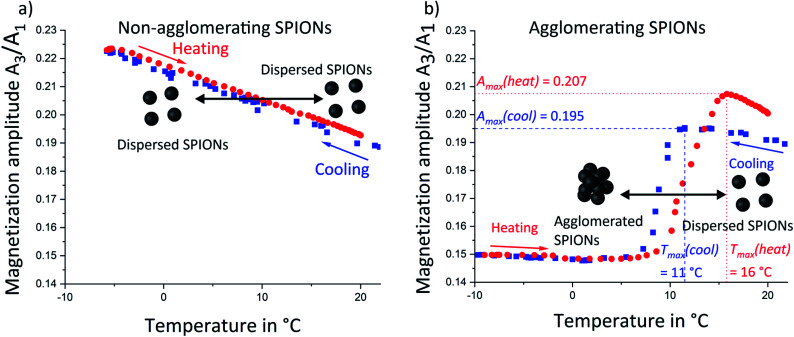
Magnetic particle spectroscopy of non-agglomerating (oleic acid modified) SPIONs (a) and agglomerating (stearic acid modified) SPIONs (b). The non-agglomerating SPIONs exhibited a linear magnetization amplitude (3^rd^ harmonic normalized to the fundamental frequency intensity) increase with decreasing temperature over the analyzed temperature range. This is consistent with temperature dependent Néel relaxation. (b) The temperature dependence of Néel relaxation is only visible at high temperatures for the agglomerating SPIONs. Decreasing temperature below 11 °C (blue squares) led to a pronounced magnetization decay that is dominated by magnetic coupling of agglomerated particles. A temperature-dependent hysteresis between the maximum magnetization amplitude during cooling *T*_max_(cool) and during heating (red circles) *T*_max_(heat) was consistent with the agglomeration hysteresis observed in SAXS. Differences in the magnetization amplitude maxima during cooling and heating (*A*_max_(cool) and *A*_max_(heat)) suggest different agglomerate structures during cooling and heating. The sharp signal drop between 5 °C and 11/16 °C is attributed to the effect of large agglomerates.

The magnetization amplitude of the 3^rd^ harmonic of this spectrum was analyzed as function of sample temperature for non-agglomerating SPIONs (Fig. 3a). It increased linearly with decreasing temperature from 25 °C to −7 °C (blue squares). Subsequent reheating (red circles) led to the inverse magnetization behavior. Increasing the temperature reduces the slope of the magnetization curve. The static magnetization amplitude of nanoparticles therefore decreases with increasing temperature. The dynamic MPS measurements at 20 kHz are sensitive to dynamic relaxation due to Néel or Brownian mechanisms. Néel relaxation is affected by the temperature dependent anisotropy constant, whereas Brownian relaxation is affected by the temperature dependent viscosity of the medium. It has been shown^24,25^ that Néel dominated relaxation of nanoparticles causes a magnetization amplitude (*A*_3_/*A*_1_) *increase* with decreasing temperature. Brownian dominated relaxation leads to a magnetization amplitude *decrease* with decreasing temperature.^24^ We observed a magnetization amplitude increase and conclude that relaxation is Néel dominated.^15,24^ The nanoparticles follow an alternating magnetic field by rotation of their atomic magnetic moments (Néel-relaxation).^20,24^ Our SPIONs are sufficiently small (TEM micrographs are provided in ESI Fig. S1†) to exhibit this mechanism.

### Magnetic characterization of thermally switchable SPIONs

The temperature-sensitive SPIONs with stearic acid shell underwent temperature-induced agglomeration that strongly affected their magnetic properties. A comparison of the magnetization amplitudes as function of higher harmonics at 25 °C and 0 °C (Fig. S8 in ESI†) revealed a faster magnetization amplitude decay at lower temperature, opposite to the trend in non-agglomerating SPIONs with oleic acid. Agglomeration apparently more than compensated for the temperature dependence of Néel-relaxation and led to an opposite temperature dependency. The temperature dependent magnetization amplitude (Fig. 3b) above 20 °C during cooling (blue squares) was similar to that of non-agglomerating reference SPIONs in the same temperature range (Fig. 3a). We conclude that well-dispersed particles above the agglomeration temperature exhibited Néel dominated relaxation, too. Temperature decrease led to a increase of magnetization amplitude until a maximum at 11 °C. Further cooling to 5 °C decreased magnetization before a slight increase in magnetization amplitude was observed below 5 °C. This trend was identical to that observed for non-agglomerating SPIONs and agglomerating SPIONs in their initial state (above 25 °C). We therefore conclude the relaxation of all particles was Néel-dominated. The trends for the 5^th^, 9^th^ and 25^th^ harmonic (Fig. S9 in ESI†) were similar, indicating that all nanoparticles (and not only a fraction thereof) followed the external magnetic field *via* Néel relaxation rather than *via* physical rotation of the particles, (Brown dominated relaxation) indicating a “magnetically homogeneous” sample.^24^ The non-agglomerating SPIONs (Fig. 3a) did not exhibit such a magnetization amplitude drop, indicating that effects correlated to the agglomeration of SPIONs were responsible for this signal variation.

Reheating of the sample (red circles) increased the magnetization amplitude, but the maximum was shifted towards a higher temperature *T*_max_ of 16 °C. This shift may indicate that de-agglomeration of SPIONs requires more thermal energy than the conservation of an initial partially dispersed state (agglomeration hysteresis). Agglomeration begins with the formation of small agglomerates throughout the volume that later merge into larger structures. De-agglomeration requires the liberation of particles that are enclosed within larger agglomerates and cannot move. Individual SPIONs can only detach at the agglomerate fringe/interface with the surrounding liquid, which leads to a shift of the de-agglomeration onset towards higher temperatures. This hysteresis was also observed in SAXS (Fig. 1a) and reported in recent work for other non-polar nanoparticles.^23^

The maximum of the magnetization amplitude (*A*_max_) of the heating curve is above the one in the cooling curve. We suggest that the magnetization amplitude of Néel-dominated SPIONs is strongly affected by particle–particle interactions (such as dipole–dipole coupling) upon agglomeration that change the magnetic relaxation with respect to an external alternating magnetic field.^26^ A magnetization amplitude decrease has been previously reported for MPS on iron oxide nanoparticles where agglomeration was induced by pH variation^27^ or by binding a marker to a target.^27,28^ This question remains ambiguous as other publications reported that dependent on the size of agglomerates and whether they are immobilized or not, a magnetization amplitude intensification is possible as well.^27^

**Fig. 4:**
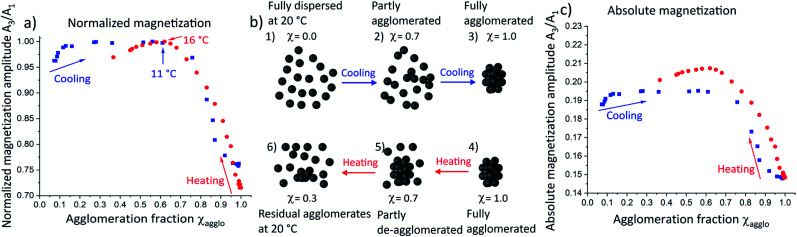
(a) The normalized magnetization amplitudes reveal that their courses are dominated by the fraction and size of agglomerates rather than by direct temperature effects. The proposed agglomerate structures are schematically illustrated (b) during cooling and heating: cooling of fully dispersed SPIONs at 20 °C (1) leads to the formation of “agglomeration nuclei”, *i.e.* small oligomers of nanoparticles (2). Only when a critical agglomeration fraction is reached, larger agglomerates form (3). Upon heating of these agglomerates (4), individual nanoparticles detach from the surfaces of agglomerates (5) yielding a mixture of freely dispersed SPIONs and relatively large agglomerates (2) which leads to a different agglomerate size distribution for cooling and heating despite of identical agglomeration fraction of *χ* = 0.7. Further heating removes agglomerates but some remain (6) at temperatures where the SPIONs had been fully dispersed initially (1). The resulting change in overall agglomerate structures is consistent with the magnetization properties observed by MPS: absolute magnetization amplitudes (c) are larger for identical agglomeration fractions during heating compared to the cooling curve.

In order to study the magnetic effect of agglomeration in more detail, we correlated the magnetization amplitude with the agglomeration fraction determined from SAXS. Magnetization amplitudes were normalized to the maximum magnetization amplitude of the heating and cooling curve respectively and plotted as a function of the agglomeration fraction *χ* (Fig. 4a). The magnetization amplitude below *χ* = 0.1 during cooling (blue squares) increased due to the temperature-dependent Néel effect as in non-agglomerating SPIONs (Fig. 3a). With progressing agglomeration up to approximately *χ* = 0.6, the magnetization amplitude increased only very slightly, indicating that agglomeration compensated for the expected temperature dependency or removed it. Increasing agglomeration to *χ* > 0.6 rapidly decreased the magnetization amplitude. We conclude that the magnetization amplitude decreased sharply only when larger agglomerates were formed.

To verify this, DLS measurements were performed at an agglomeration fraction of *χ* = 0.75 at 10 °C (see Fig. S10 in ESI†). A very slight increase of the maximum hydrodynamic diameter of only a few nanometers was observed, confirming the hypothesis that no large agglomerates had formed up to this point. A schematic illustration of the possible agglomerate structure at 10 °C is provided in Fig. 4b2. We propose that individual nanoparticles (Fig. 4b1) form “agglomeration nuclei”, *i.e.* small oligomers of nanoparticles throughout the volume (Fig. 4b2), that do not combine into larger agglomerates yet (Fig. 4b3). The oligomers affect magnetization far less than larger agglomerates, where the magnetic moments strongly couple as described in the Stoner–Wohlfarth and Néel relaxation models.^29,30^ The magnetization of large agglomerates lags behind the external field, which causes a decrease in magnetization amplitude.

Heating large agglomerates (Fig. 4a, red circles and schematically illustrated in Fig. 4b4) led to a continuous magnetization amplitude increase up to *χ* = 0.6, as individual SPIONs continuously detached from the large agglomerates. Note that a maximum in magnetization amplitude occurred at the same agglomeration fraction as in the cooling curve, although these occur at different temperatures (11 °C and 16 °C). We conclude that the magnetization amplitude is strongly dominated by the agglomeration fraction and that temperature effects are negligible.

We compared absolute magnetization amplitudes as a function of agglomeration fraction (Fig. 4c), too. Heating (red circles) of the agglomerated SPIONs towards smaller agglomeration fraction led to an elevated magnetization amplitude that exceeded the maximal value encountered during cooling. We thus propose that fewer, larger agglomerates are present during heating (Fig. 4b5). They increase the magnetization amplitude compared to the larger number of smaller agglomerates that form during cooling (Fig. 4b2). Further heating towards an agglomeration fraction below *χ* = 0.6, caused the magnetization amplitude to decline (Fig. 4c). The magnetization amplitude at 20 °C was still greater than that at the same temperature of the cooling curve. The only difference between the two states was the incomplete de-agglomeration upon heating (*χ* = 0.37, Fig. 4b6) compared to freely dispersed SPIONs during cooling. The remaining agglomerates increased the magnetization amplitude compared to fully dispersed nanoparticles.

We conclude that small agglomerates of SPIONs (as present during initial cooling) only slightly alter the magnetic properties while large agglomerates (at low temperatures) reduce the magnetization amplitude significantly. An intermediate size/structure present during heating increases the magnetization amplitude.

## Conclusion

Temperature induced agglomeration with organic ligand shells was demonstrated for superparamagnetic iron oxide nanoparticles. The state of agglomeration was reversibly adjustable and changed the magnetic properties of iron oxide nanoparticles as indicated *in situ* by magnetic particle spectroscopy during temperature variation. It was revealed that a non-linear relation between agglomeration state and magnetic properties exists. The effect of temperature-dependent agglomeration on magnetization was much stronger than the direct effect of temperature on dispersed particles. If transferred to aqueous systems, this might be interesting for improving nanoparticle properties in applications such as 3D imaging thermometry or hyperthermia.

## Conflicts of interest

There are no conflicts to declare.

## Supplementary Material

NA-003-D1NA00159K-s001
