# Supplementary material for: Reversible magnetism switching of iron oxide nanoparticle dispersions by controlled agglomeration
Source: Nanoscale Adv. 2021 Mar 30;3(10):2822–9. doi: 10.1039/d1na00159k (PMC9417811; doi:10.1039/d1na00159k)
Supplement: NA-003-D1NA00159K-s001 [file NA-003-D1NA00159K-s001.pdf]

Electronic Supplementary Material (ESI) for Nanoscale Advances.
This journal is © The Royal Society of Chemistry 2021

SUPPORTING INFORMATION

Reversible magnetism switching of iron oxide nanoparticle dispersions by controlled agglomeration

Stephan Müssig[a], Björn Kuttich[b], Florian Fidler[c], Daniel Haddad[c], Susanne Wintzheimer[a], Tobias Kraus[b,d], Karl Mandel*[a,e]*

a) Department of Chemistry and Pharmacy, Friedrich-Alexander University Erlangen-Nürnberg (FAU), Egerlandstraße 1, 91058 Erlangen, Germany

b) INM − Leibniz-Institute for New Materials, Campus D2 2, 66123 Saarbrücken, Germany.

c) Magnetic Resonance and X-ray Imaging Department, Development Center X-ray Technology, Fraunhofer Institute for Integrated Circuits IIS, Am Hubland, D-97074 Würzburg, Germany.

d) Colloid and Interface Chemistry, Saarland University, 66123 Saarbrücken, Germany

e) Fraunhofer-Institute for Silicate Research ISC, Neunerplatz 2, 97082 Würzburg, Germany

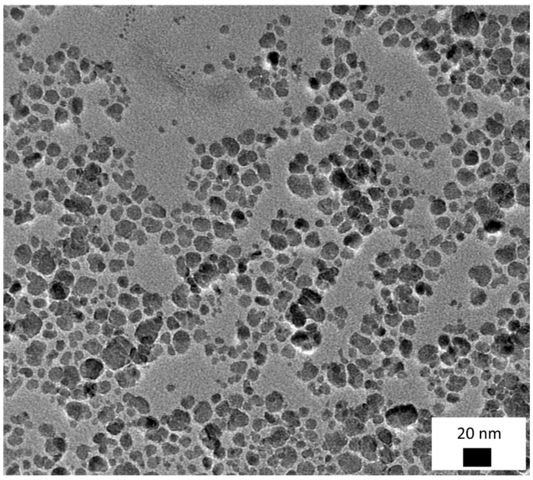

Figure S1: Transmission electron microscopy (TEM) micrograph of superparamagnetic iron oxide nanoparticles stabilized by an oleic acid ligand shell.

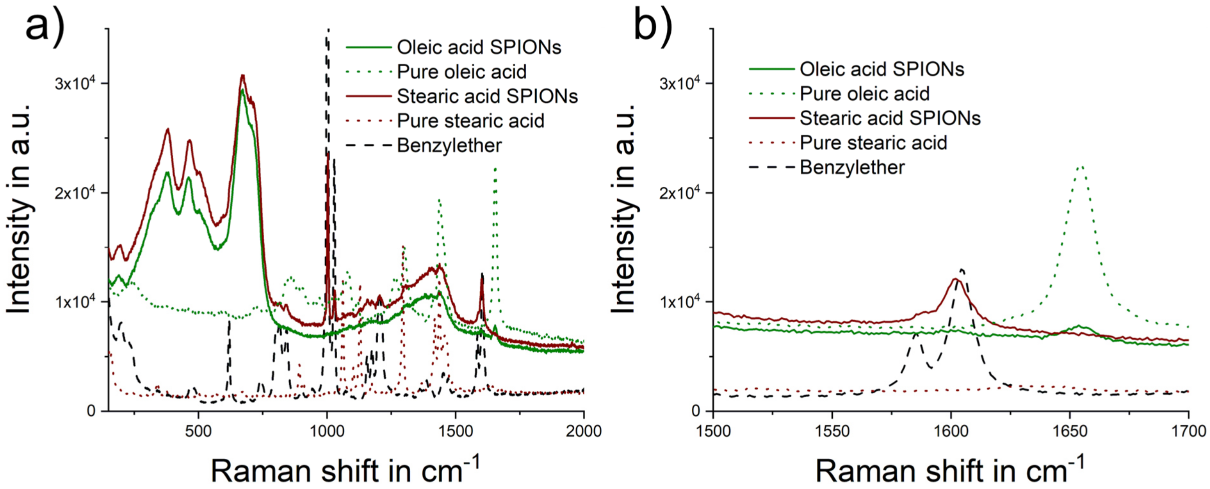

Figure S2: Raman spectra (a) of dried SPION dispersions stabilized by stearic or oleic acid, respectively. Around 1650 cm^{-1} (b) the oleic acid C = C stretching band is clearly visible. Ligand exchange with stearic acid removes this band. Benzylether was used as a solvent during ligand exchange and is detected for the stearic acid sample as well. This is a minor component that likely does not influence the ligand properties of stearic acid.

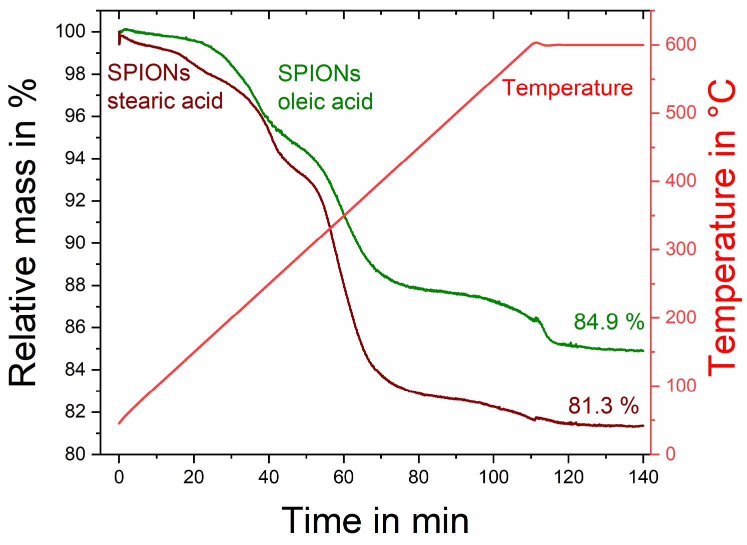

Figure S3: Thermogravimetric analysis of SPIONs with oleic acid before ligand exchange (green line) and after ligand exchange with stearic acid (brown line). After decomposition up to 600 °C, the residual masses are 84.9 % and 81.3 % for oleic and stearic acid, respectively. Note that residual benzylether was detected by Raman spectroscopy (Fig. S2) in case of the stearic acid. Thus, the lower residual mass is not only due to ligand decomposition but also due to decomposition of an unknown amount of solved benzylether. Thus, we conclude that the amount of organic material and thus the ligand density of stearic acid and oleic acid on the surface of SPIONs are similar are no multiple ligand layer have formed.

Analysis of small angle X-ray scattering data

Small angle scattering data is measured as intensity in dependence of the modulus of the scattering vector $\vec{q}$. Exemplary scattering curves after background subtraction and normalization to absolute intensities for SPIONS stabilized by stearic acid are shown on the left hand side of figure S4. At the highest depicted temperatures data consists of a form factor only, showing no scattering peak at intermediate q-values and an intensity plateau at small q. Upon cooling a structure factor emerges leading to a weak peak feature around q = 0.04 Å^{-1} and an increasing intensity at low q. Due to the rather high polydispersity the scattering peak stemming from regular particle-particle distances within the agglomerates is very broad. A precise fitting model for this system is difficult to obtain and lies beyond the scope of this manuscript. We therefore analyze the data by means of an effective structure factor depicted exemplarily on the right hand side of figure S4. The effective structure factor is obtained by dividing the scattered intensity by the form factor obtained by fitting a polydisperse spherical model to non–agglomerated high temperature data. As shown on the right hand side of figure S4, even in the structure factor representation the correlation peak is broad and weak due to the high polydispersity. We therefor focus on the emerging structure factor minimum which is more pronounced and therefore analysis becomes more robust.

Nanoparticle agglomeration does not occur at a sharp transition temperature but a smooth, smeared transition is observed. This is caused by different inhomogeneities like size polydispersity, ligand density differences and local variations in particle concentration. At any given temperature an equilibrium system is observed with a certain ratio of nanoparticles being freely dispersed while other particles have already agglomerated. Scattering intensity of these different species add up incoherently leading to a simple sum of intensity scattered from

dispersed and agglomerated samples. For dispersed particles the structure factor is equal to unity independent of the scattering vector, while agglomerated particles exhibit a distinct structure factor depending on the precise structure of the agglomerate. The effective structure factor shown in figure S4 contains information from both species and a careful evaluation of its deviation from unity allows do determine the ratio of dispersed and agglomerated particles, leading to the agglomeration fraction χ_{agglo}.

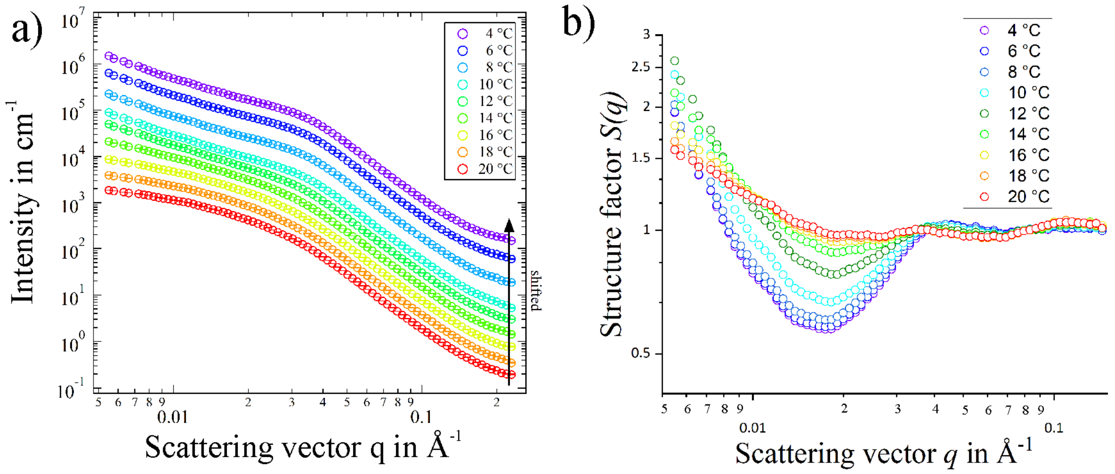

Figure S4: Temperature dependent SAXS data for stearic acid stabilized SPIONS in decane (a). Curves are shifted vertically for clarity. b) Effective structure factor deduced from the SAXS data by division of a polydisperse spherical form factor model. Emergence of a strong minimum around 0.02 Å^{-1} is a clear indication of agglomeration.

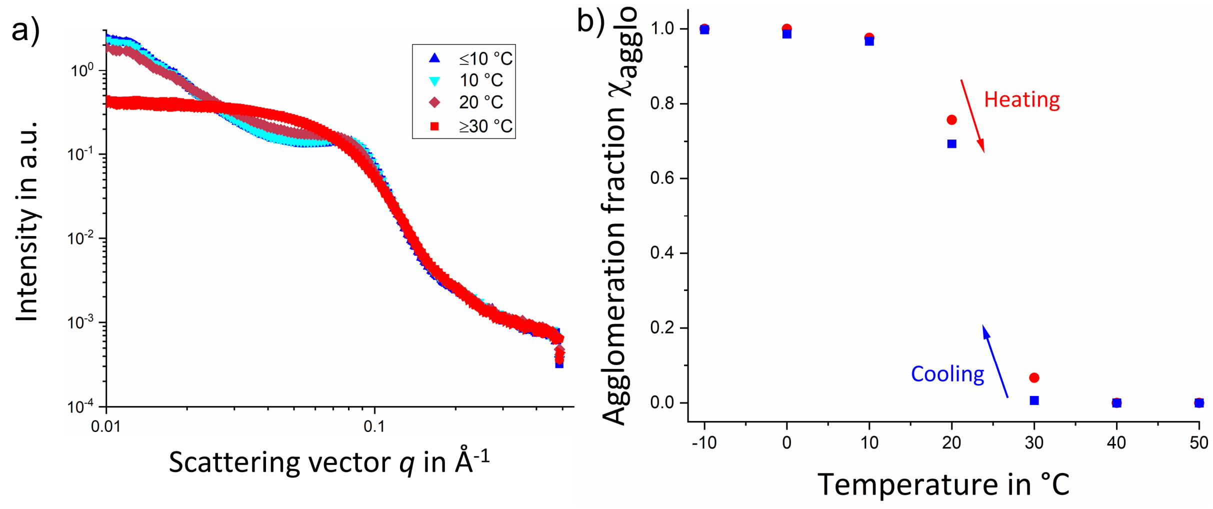

Figure S5: Temperature dependent SAXS data for behenic acid stabilized SPIONS in toluene (a). The calculated agglomeration fraction (b) indicates complete agglomeration upon cooling and a fully reversible de-agglomeration after subsequent heating above 40 °C.

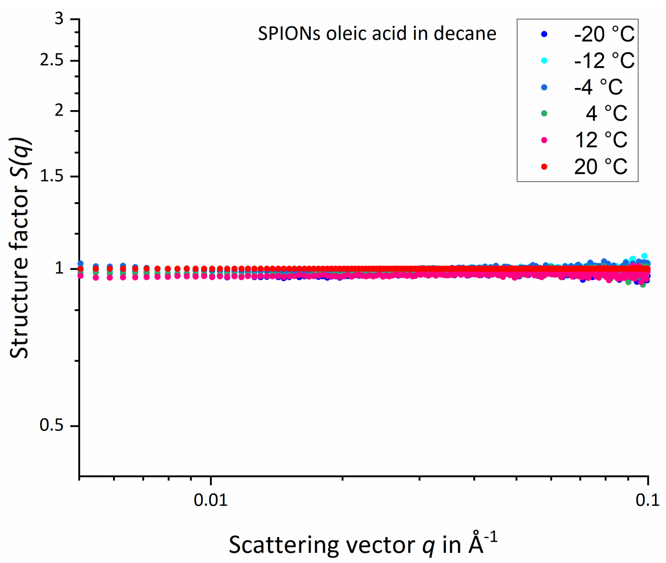

Figure S6: Temperature dependent structure factor for oleic acid stabilized SPIONS in decane deduced from SAXS data. No agglomeration is visible.

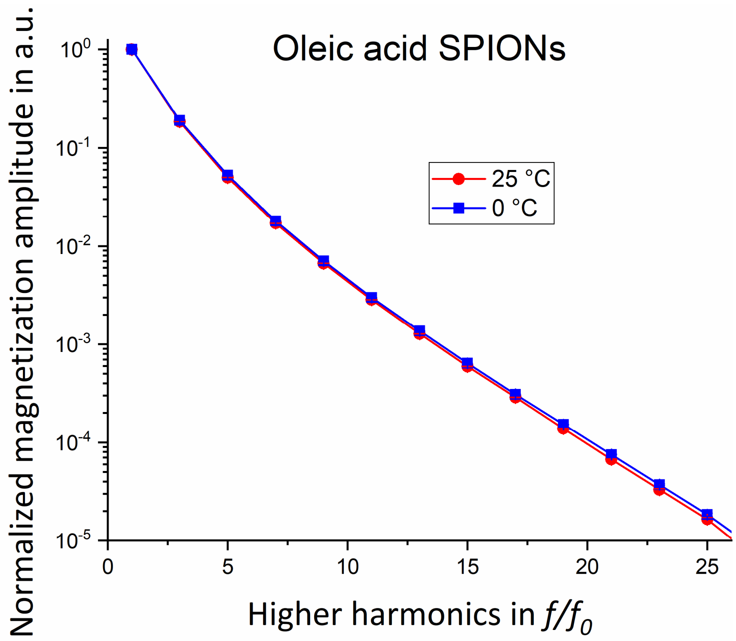

Figure S7: Magnetization amplitudes from Magnetic Particle Spectroscopy (MPS) normalized to the corresponding fundamental intensity as a function of the higher harmonics of the employed SPIONs with oleic acid as ligand at 25 °C (red circles) and 0 °C (blue squares) when no agglomeration occurs.

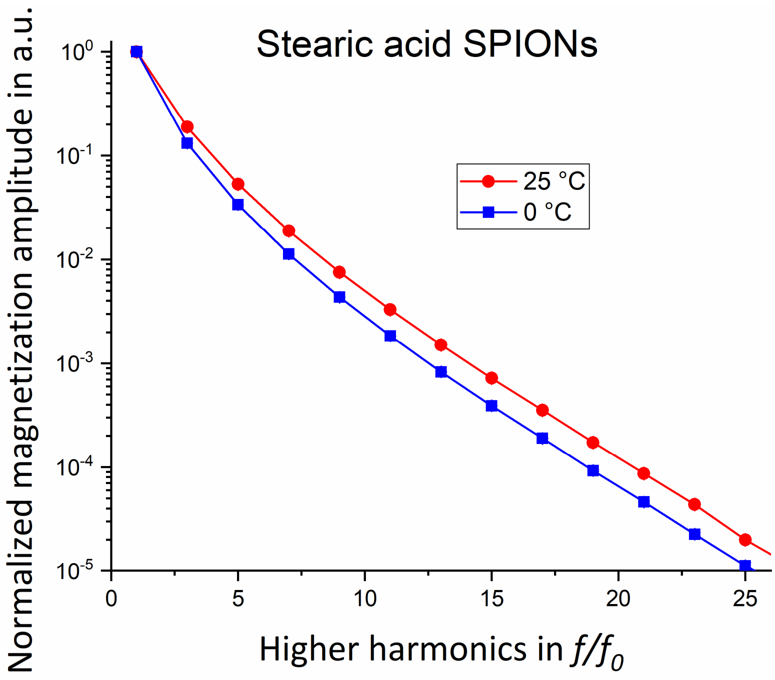

Figure S8: Magnetization amplitudes from MPS normalized to the corresponding fundamental intensity as a function of the higher harmonics of the employed SPIONs with stearic acid as ligand at 25 °C (red circles) and 0 °C (blue squares) upon agglomeration.

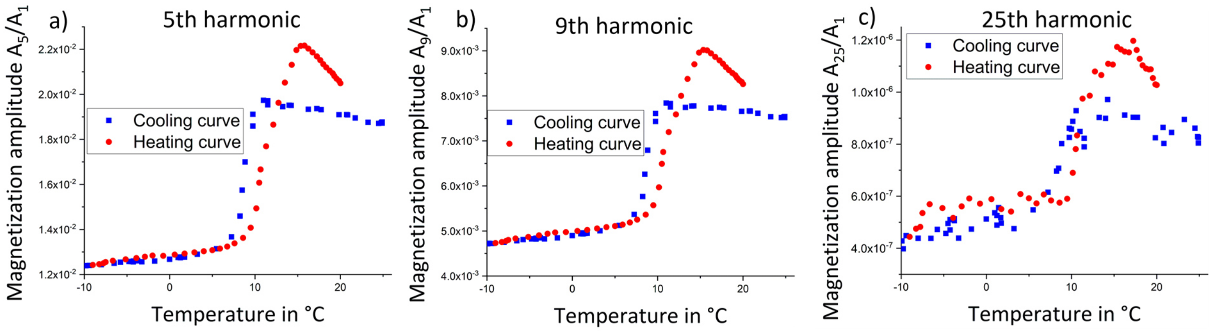

Figure S9: Magnetization amplitude intensities of the 5th (a), 9th (b) and 25th (c) higher harmonic from MPS normalized to the corresponding fundamental intensity as a function of temperature. The identical trend was observed for all harmonics, indicating similar temperature influences on the entire sample.

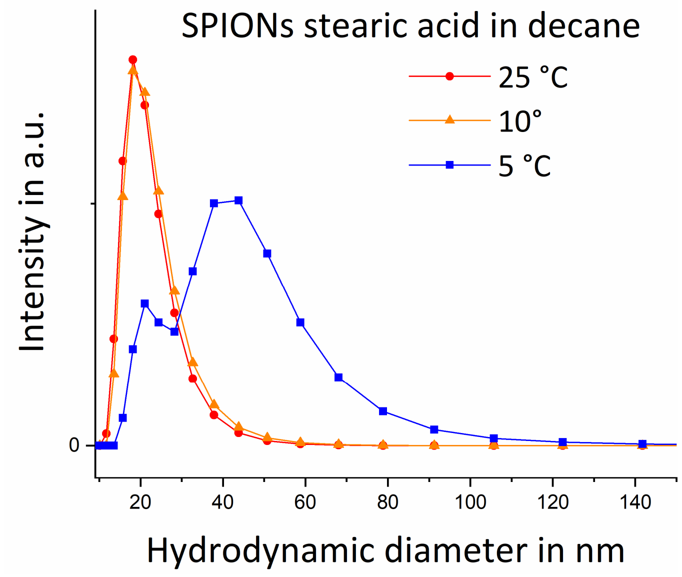

Figure S10: Dynamic Light Scattering (DLS) of agglomerating SPIONs with stearic acid as ligand at 25 °C (red circles), 10 °C (orange triangles) and 5 °C (blue squares) in decane. Between 25 °C and 10 °C the hydrodynamic diameter shifted slightly towards larger sizes while at 5 °C a large fraction of considerably larger agglomerates of SPIONs was present.
